# Supplementary material for: Cytokinin functions as an asymmetric and anti-gravitropic signal in lateral roots
Source: Nat Commun. 2019 Aug 6;10:3540. doi: 10.1038/s41467-019-11483-4 (PMC6684572; doi:10.1038/s41467-019-11483-4)
Supplement: Supplementary file 1 — Supplementary Information [file 41467_2019_11483_MOESM1_ESM.pdf]

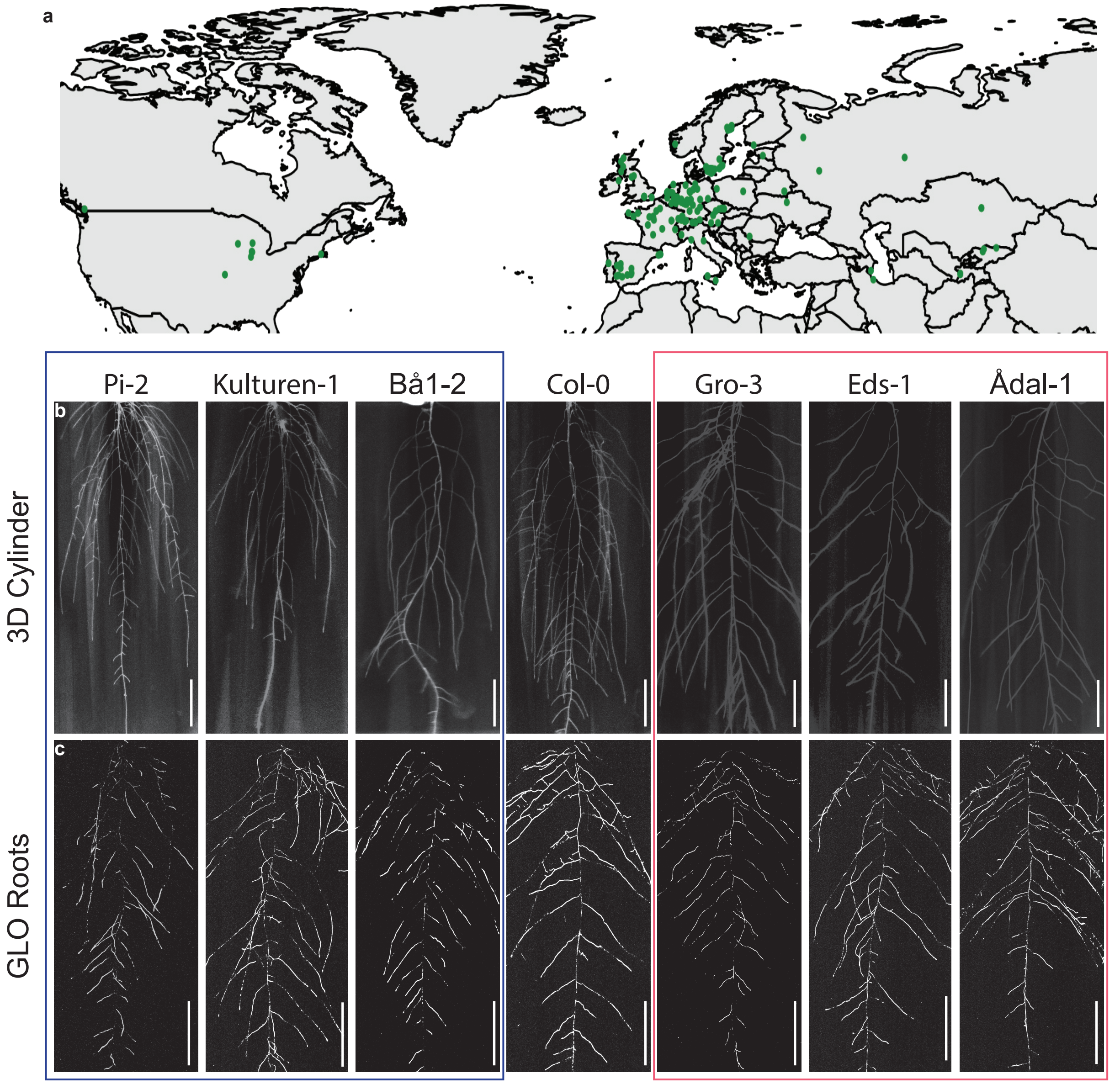

Supplementary Fig. 1. Accessions used in this study and representative root system images of selected accessions.
(a) Geographical distribution of natural Arabidopsis thaliana accessions used in this study. The distribution of accessions is visualized by R package “rworldmap”.
(b) 10-day old accessions grown in 3D agar cylinders. Scale bars, 1 cm.
(c) 20-day old accessions grown in soil. Scale bars, 5 cm.
(b)-(c) Representative images are shown. Experiments were repeated at least three times.

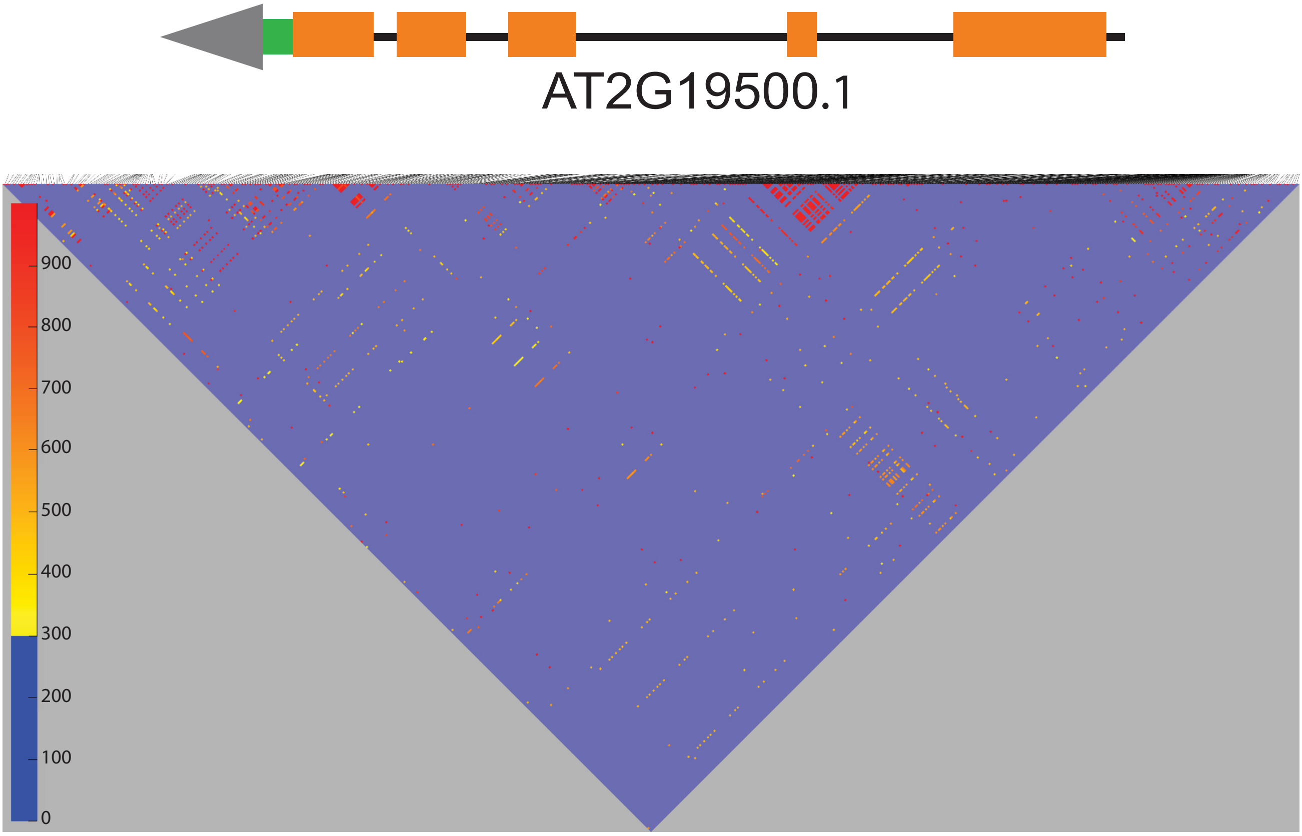

Supplementary Fig. 2. Calculation of linkage disequilibrium by pairwise comparison of 500 SNPs.
r2 value is scaled and color-coded (blue to red) from 0 to 1 (low to high association). Underlying code can be found at github https://github.com/timeu/PyGWAS/blob/master/pygwas/core/genotype.py#L59).

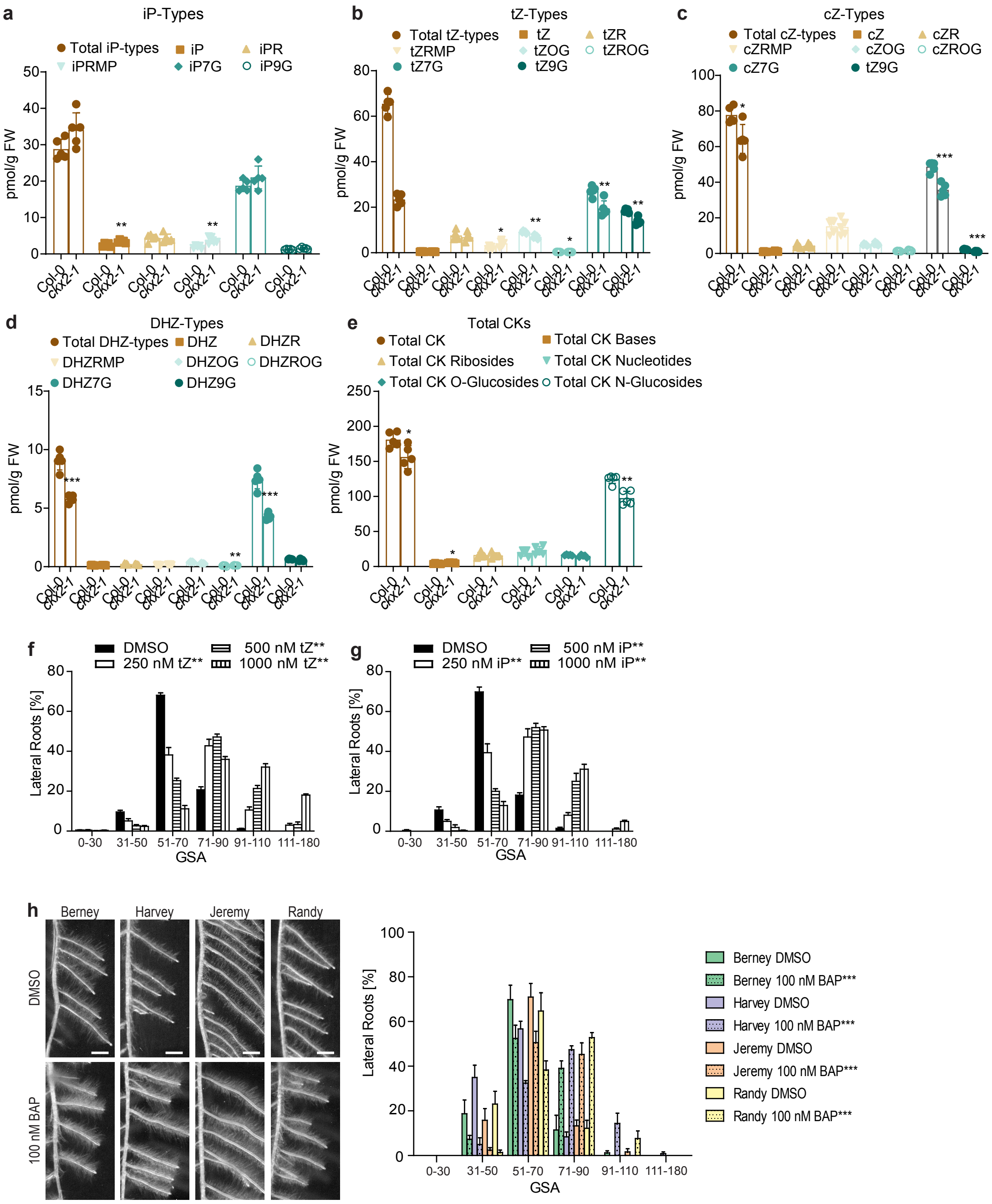

Supplementary Fig. 3. Influence of cytokinin on GSA of lateral roots.

(a)-(e) Quantification of different CK forms (nucleotides (precursors), ribosides (transported forms), free bases (active forms), and O-/N-glucosides (reversible/irreversible inactivated storage forms) in *Col-0* wild type and *ckx2-1* mutant roots. (a) iP-Types, (b) tZ-Types, (c) cZ-Types, (d) DHZ-Types, (e) Total CKs.

(f)-(g), GSA distributions of DMSO and (f) trans-zeatin (tZ)-treated or (g) isopentenyladenine (iP)-treated *Col-0* wild type seedlings.

(h) Representative images and GSA distributions of four different untreated and BA-treated winter oilseed rape (*Brasica napus* L.) genotypes.

(a)-(e) One-way ANOVA analysis P-values: * P < 0.05, ** P < 0.01, *** P < 0.001. Mean ± SD, n = 5 extractions.

(f)-(h) Kolmogorov-Smirnov test P-values: * P < 0.05, ** P < 0.01, *** P > 0.001 (compared to DMSO or *Col-0*, respectively). Mean ± SEM, for *A. thaliana*: n = 5 plates (16 seedlings with 80-160 LRs per plate), for oilseed rape: n = 3 seedlings with 25-50 LRs per seedling). Experiments were repeated at least three times.

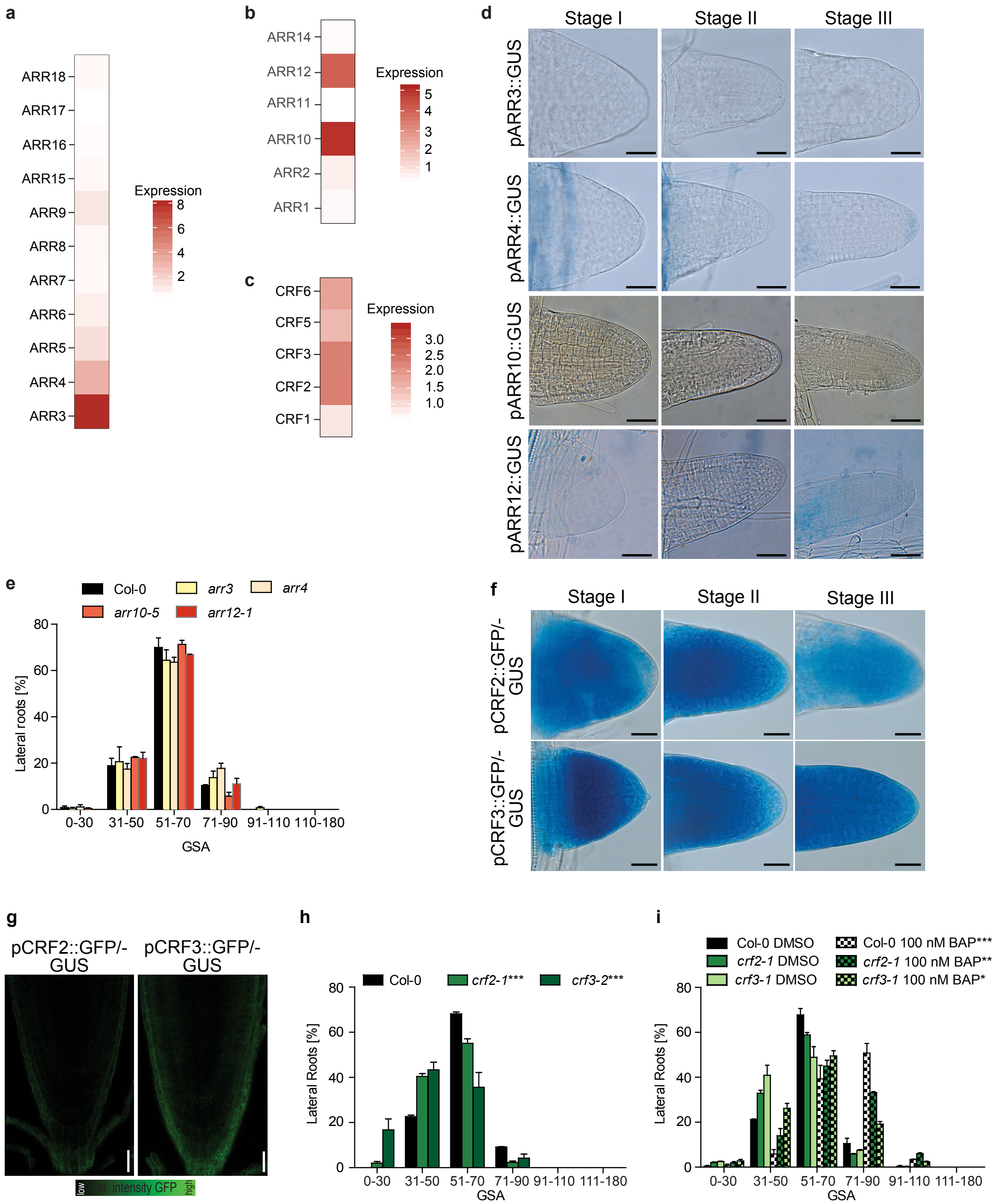

a
ARR18
ARR17
ARR16
ARR15
ARR9
ARR8
ARR7
ARR6
ARR5
ARR4
ARR3
Expression
8
6
4
2
b
ARR14
ARR12
ARR11
ARR10
ARR2
ARR1
Expression
5
4
3
2
1
c
CRF6
CRF5
CRF3
CRF2
CRF1
Expression
3.0
2.5
2.0
1.5
1.0
d
Stage I
Stage II
Stage III
pARR3::GUS
pARR4::GUS
pARR10::GUS
pARR12::GUS
e
Col-0
arr3
arr4
arr10-5
arr12-1
Lateral roots [%]
80
60
40
20
0
0-30
31-50
51-70
71-90
91-110
110-180
GSA
f
Stage I
Stage II
Stage III
pCRF2::GFP/-GUS
pCRF3::GFP/-GUS
g
pCRF2::GFP/-GUS
pCRF3::GFP/-GUS
low
rel. intensity GFP
high
h
Col-0
crf2-1***
crf3-2***
Lateral Roots [%]
80
60
40
20
0
0-30
31-50
51-70
71-90
91-110
111-180
GSA
i
Col-0 DMSO
crf2-1 DMSO
crf3-1 DMSO
Col-0 100 nM BAP***
crf2-1 100 nM BAP**
crf3-1 100 nM BAP*
Lateral Roots [%]
80
60
40
20
0
0-30
31-50
51-70
71-90
91-110
111-180
GSA

Supplementary Fig. 4. Role of Arabidopsis response regulators (ARRs) and cytokinin response factors (CRFs) in the GSA establishment in LRs.
(a) Expression of type-A ARRs in lateral roots. Data from[25].
(b) Expression of type-B ARRs in lateral roots. Data from[25].
(c) Expression of CRFs in lateral roots. Data from[25].
(d) GUS staining of pARR3::GUS, pARR4::GUS, pARR10::GUS and pARR12::GUS. Scale bars, 25 µm.
(e) GSA distribution of *Col-0* wild-type, *arr3*, *arr4*, *arr10-5* and *arr12-1* mutants. Kolmogorov-Smirnov test. Mean ± SEM, n = 5 plates (16 seedlings with 25-120 LRs per plate).
(f) Representative images after GUS staining of pCRF2::GFP-GUS and pCRF3::GFP-GUS in stage I-III LRs. Scale bars, 10 µm.
(g) Representative images of pCRF2::GFP-GUS and pCRF3::GFP-GUS in the main root tip. Scale bars, 25 µm.
(h) GSA distribution of *Col-0* wild type and crf single mutants.
(i) GSA distribution of DMSO and BAP treated *Col-0* wild type, *crf2-1* and *crf3-1* single mutants.
(h) – (i) Kolmogorov-Smirnov test P-values: * $P < 0.05$, ** $P < 0.01$, *** $P > 0.001$ (compared to DMSO or *Col-0*). Mean ± SEM, n = 5 plates (16 seedlings with 20-180 LRs per plate).
(d)-(i) Experiments were repeated three times.

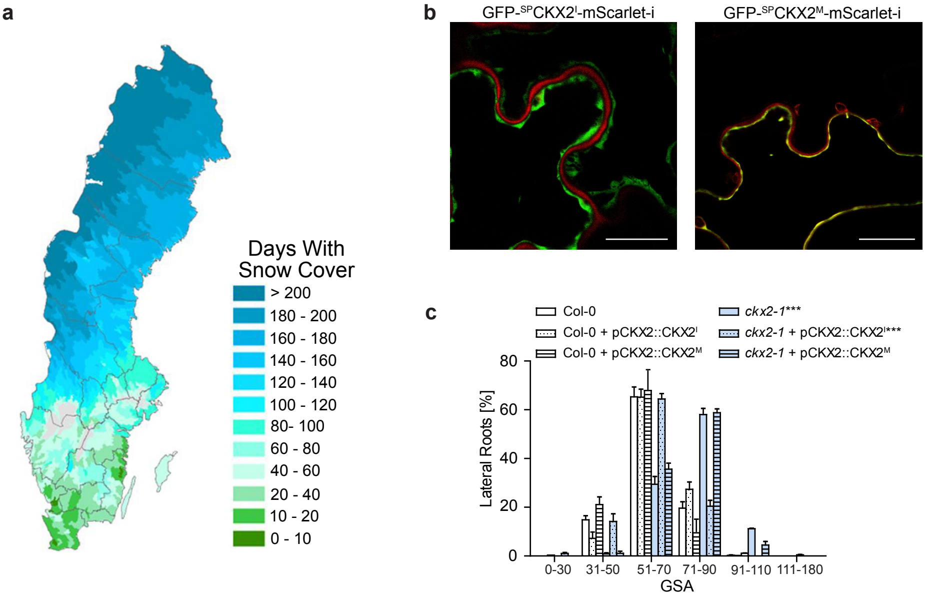

Supplementary Figure 5. Snow cover in Sweden and characterization of $CKX2^{I}$ and $CKX2^{M}$.

Supplementary Fig. 5. Snow cover in Sweden and characterization of CKX2I and CKX2M.

(a) Average number of days with snow cover in Sweden between 1961-1990. Source: https://bit.ly/2UmLAeT

(b) Localization of GFP-$^{SP}CKX2^{I}$-mScarlet and GFP-$^{SP}CKX2^{M}$-mScarlet. Tobacco leaves were infiltrated with Agrobacterium tumefaciens containing constructs and the expression of CKX2 fluorescent protein was visualized by confocal laser scanning microscopy three days after infiltration. Scale bar, 25 µm. Representative images are shown.

(c) GSA distributions of *Col-0* and *ckx2-1* as well as $CKX2^{I}$ and $CKX2^{M}$ expressing lines in both backgrounds. Kolmogorov-Smirnov test P-value: *** $P < 0.001$ (compared to Col-0). Mean ± SEM, n = 5 plates (16 seedlings with 80-160 LRs per plate).

(b)-(c) Experiments were repeated at least three times.

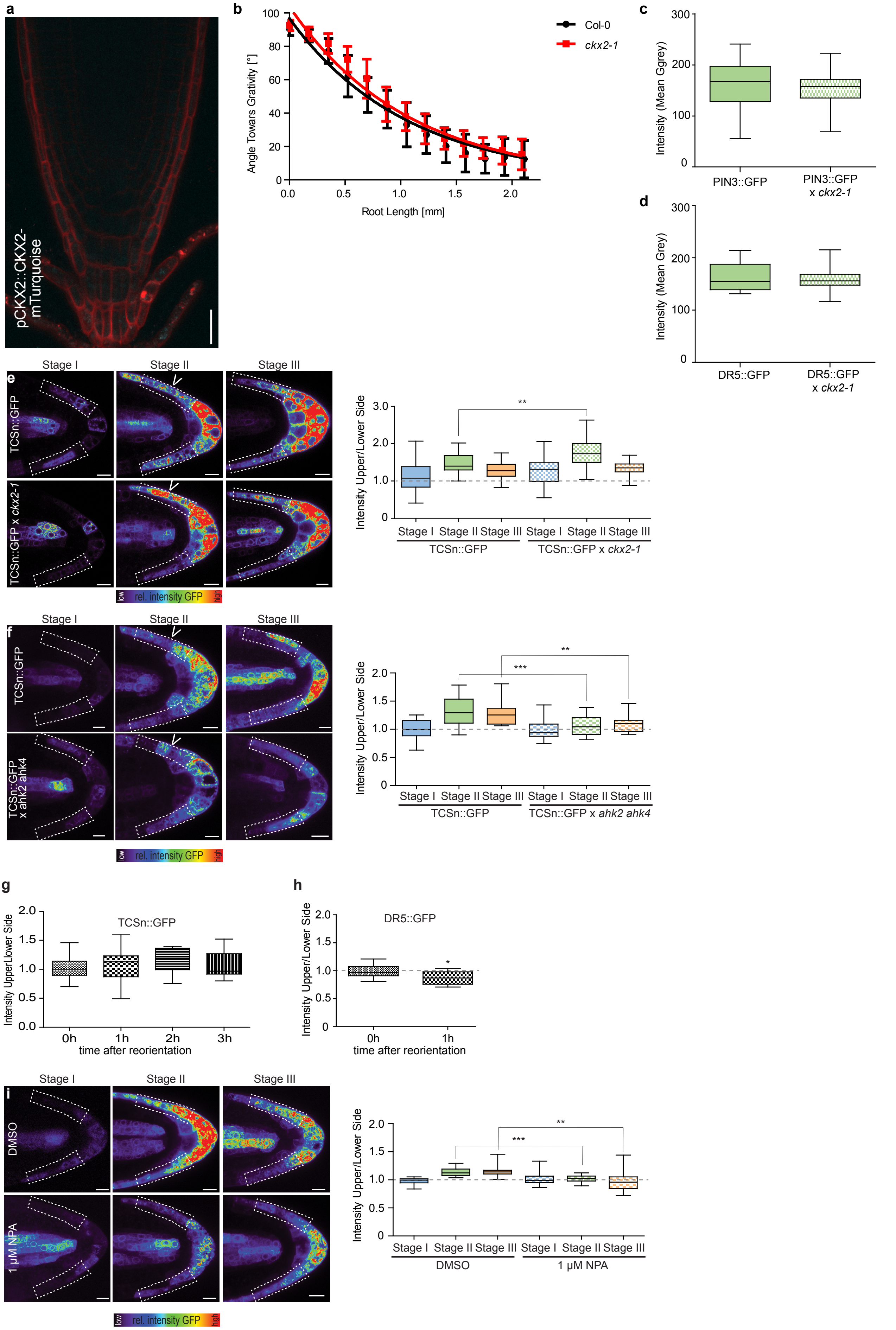
a
pCKX2::CKX2-mTurquoise
b
Col-0
ckx2-1
Angle Towars Gravity [°]
Root Length [mm]
c
Intensity (Mean Ggrey)
PIN3::GFP
PIN3::GFP x ckx2-1
d
Intensity (Mean Grey)
DR5::GFP
DR5::GFP x ckx2-1
e
Stage I
Stage II
Stage III
TCSn::GFP
TCSn::GFP x ckx2-1
rel. intensity GFP
low
high
Intensity Upper/Lower Side
**
f
TCSn::GFP
TCSn::GFP x ahk2 ahk4
***
**
g
TCSn::GFP
Intensity Upper/Lower Side
0h
1h
2h
3h
time after reorientation
h
DR5::GFP
*
i
DMSO
1 µM NPA

Supplementary Fig. 6. Localization of CKX2 in the main root tip and characterization of cytokinin responses in *ckx2-1* mutants.
(a) Representative images of pCKX2::CKX2-mTurquoise in the main root. Propidium Iodide (PI) was used for counterstaining. Scale bar, 25 µm.
(b) Gravitropic response of dark grown *Col-0* and *ckx2-1*. Mean ± SD, n = 10-15 individual roots.
(c)-(d) Signal quantification of (b) pPIN3::PIN3-GFP and (c) DR5::GFP in stage II LRs.
(e)-(f) Representative images and quantifications of (a) TCSn::GFP in *Col-0* wild type and *ckx2-1*, (b) TCSn::GFP in *Col-0* wild type and *ahk2 ahk4* in stage I – III LRs. Scale bar, 10 µm.
(g) Signal quantification of TCSn::GFP in the main root after gravity stimulation.
(h) Signal quantification of DR5::GFP in the main root after gravity stimulation (positive control for (g).
(i) Representative images and quantifications of TCSn::GFP in *Col-0* wild type after treatment with DMSO or 1 µM NPA for 24h in stage I – III LRs. Scale bar, 10 µm.
(c)-(d), (e)-(i) One-way ANOVA P-values: * $P < 0.05$, ** $P< 0.01$, *** $P< 0.001$. Horizontal lines show the medians; box limits indicate the 25th and 75th percentiles; whiskers extend to the min and max values. n = 10-40 individual LRs or main roots.
(a)-(i)Experiments were repeated at least three times.

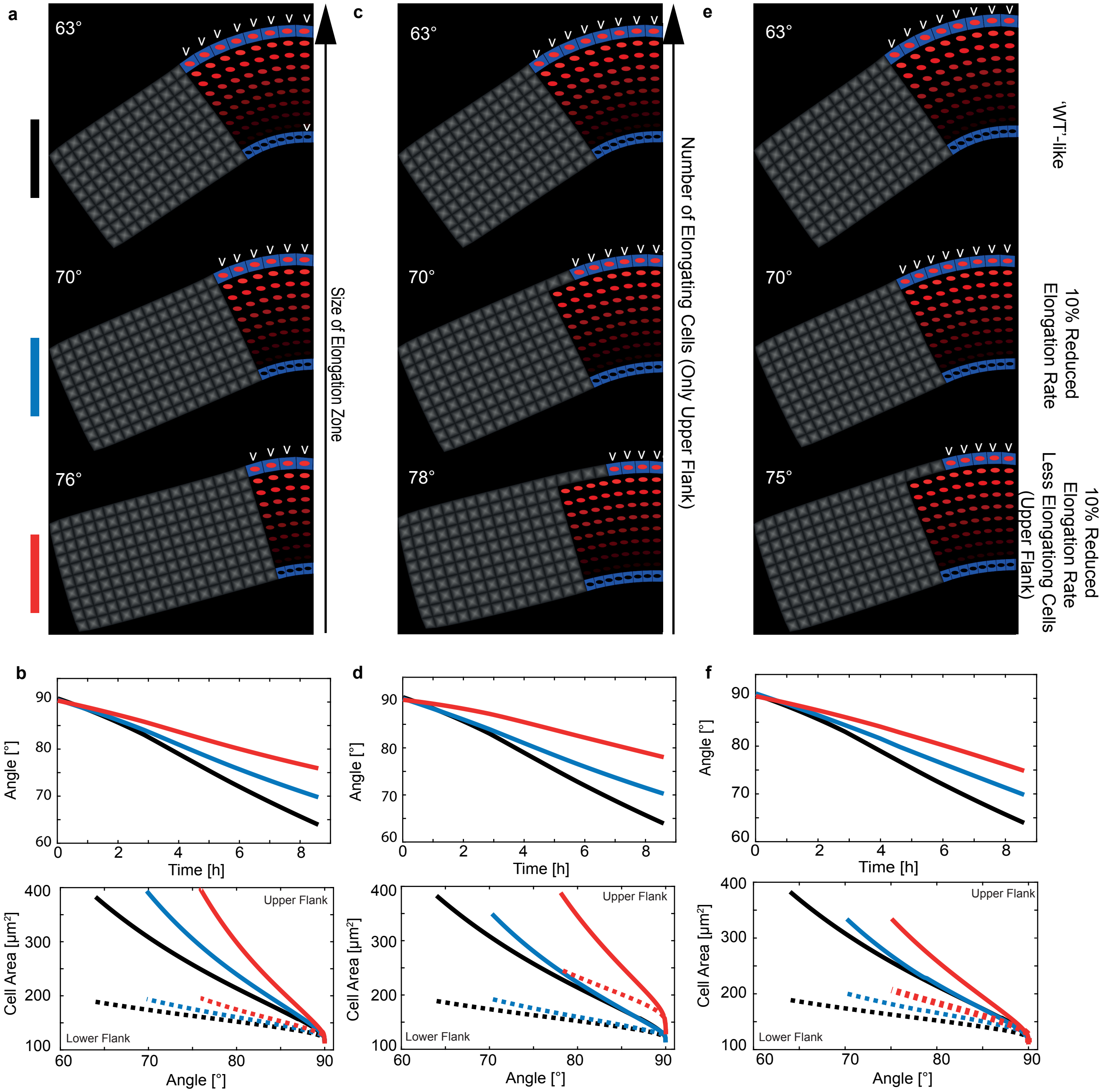
a
63°
70°
76°
Size of Elongation Zone
c
63°
70°
78°
Number of Elongating Cells (Only Upper Flank)
e
63°
70°
75°
'WT'-like
10% Reduced Elongation Rate
10% Reduced Elongation Rate Less Elongating Cells (Upper Flank)
b
d
f
Angle [°]
Time [h]
Cell Area [µm²]
Upper Flank
Lower Flank
Angle [°]

Supplementary Figure 7. Simulations of dynamic computer model of lateral root predict an inverse relation between set-point angle and the number of cells in elongation zone or the size of elongation zone.
(a)-(b) A positive impact of the size of elongation zone on the GSA angle. The longer the elongation zone (white arrow heads) the larger the GSA angle is.
(c)-(d) The number of cells on the upper flank has strong negative effect on the GSA angle. Also, model predicts that the fold difference between the size of elongating cell on the upper versus lower LR root flanks may decreases with strongly reduced number of cells, largely due to mechanical constrains present on the upper root flank.
(e)-(f) Upper panel, corresponds Fig. 7b. Middle panel, 10% decrease in elongation rate only on the upper root flank (white arrow heads). Lower panel, combination of 10% decrease in elongation rate and number of cells (shorter meristem) only on the upper root flank. Each simulation represents LR status after 9h of dynamic elongation.
(b), (d, (f) Upper panel: Time evolution of set-point angle corresponding to respective scenarios. Lower panel: Predicted GSA angle plotted against the predicted cell size.
Colours at the left side (a), (c), (e) matches simulations in (b), (d), (f). Simulations represent LR status after 8h of dynamic elongation. Other symbols are as in Figure 7.

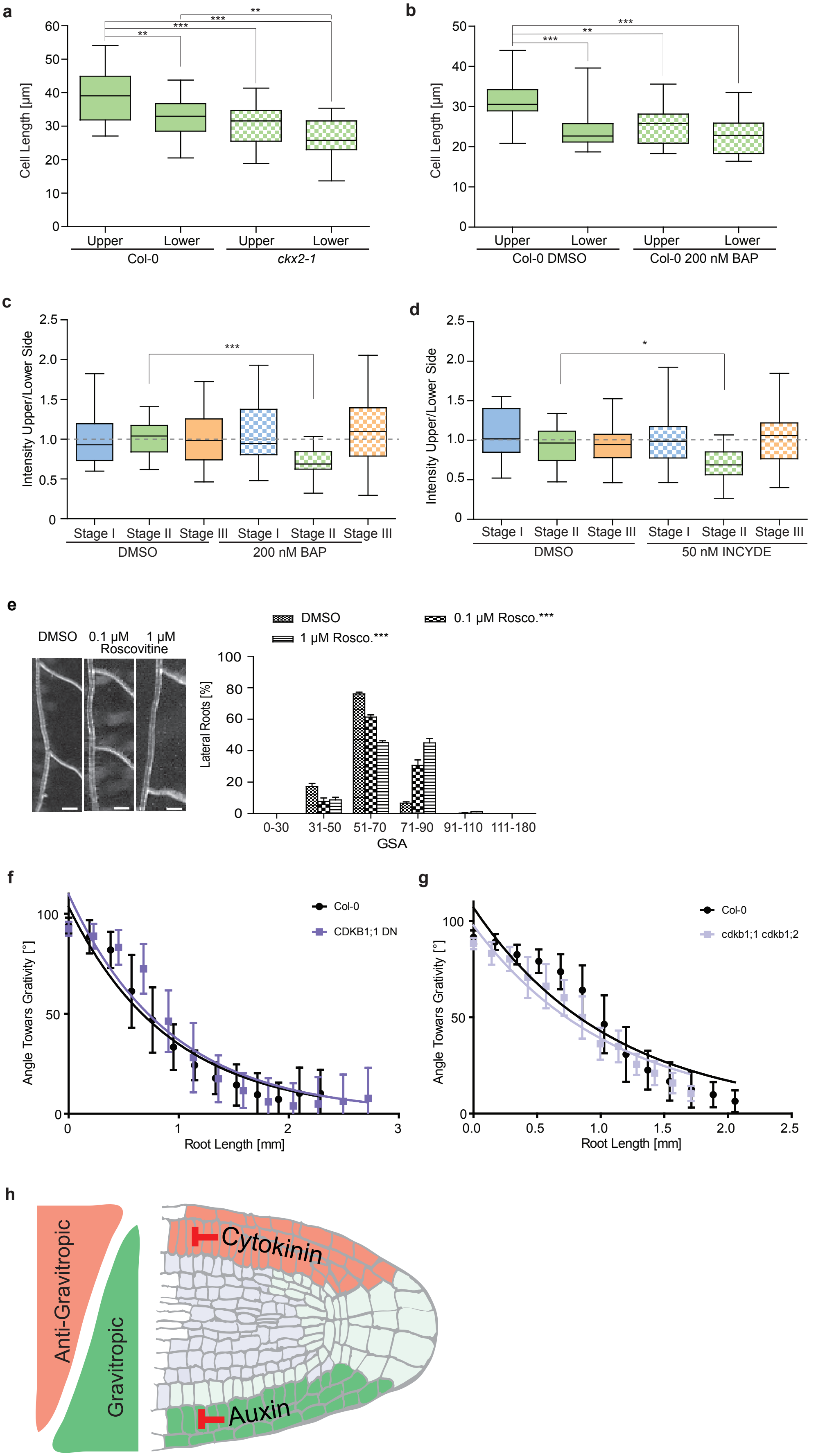
a
Cell Length [µm]
60
50
40
30
20
10
0
**
***
***
**
Upper
Lower
Upper
Lower
Col-0
ckx2-1
b
Cell Length [µm]
50
40
30
20
10
0
***
**
***
Upper
Lower
Upper
Lower
Col-0 DMSO
Col-0 200 nM BAP
c
Intensity Upper/Lower Side
2.5
2.0
1.5
1.0
0.5
0
***
Stage I
Stage II
Stage III
Stage I
Stage II
Stage III
DMSO
200 nM BAP
d
Intensity Upper/Lower Side
2.5
2.0
1.5
1.0
0.5
0
*
Stage I
Stage II
Stage III
Stage I
Stage II
Stage III
DMSO
50 nM INCYDE
e
DMSO
0.1 µM
1 µM
Roscovitine
DMSO
0.1 µM Rosco.***
1 µM Rosco.***
Lateral Roots [%]
100
80
60
40
20
0
0-30
31-50
51-70
71-90
91-110
111-180
GSA
f
Angle Towars Grativity [°]
100
50
0
Col-0
CDKB1;1 DN
0
1
2
3
Root Length [mm]
g
Angle Towars Grativity [°]
100
50
0
Col-0
cdkb1;1 cdkb1;2
0.0
0.5
1.0
1.5
2.0
2.5
Root Length [mm]
h
Anti-Gravitropic
Gravitropic
Cytokinin
Auxin

Supplementary Figure 8. Cytokinin-dependent interference with cell elongation and division rates defines angular growth of lateral roots.

(a)-(b) Quantification of first two elongated cells in the upper and lower flank of stage II lateral roots of (a) *Col-0* and *ckx2-1* and (b) after treatment with DMSO or 200 nM BAP for 24h.

(c)-(d) Quantification of pCycB1;1::GUS after treatment with (c) DMSO or 200 nM BAP or (d) DMSO or 50 nM INCYDE for 24h.

(a)-(d) One-way ANOVA P-values: * $P < 0.05$, *** $P < 0.001$. Horizontal lines show the medians; box limits indicate the 25th and 75th percentiles; whiskers extend to the min and max values. (a)-(b) n = 10-15 individual LRs, (c)-(d) n= 15-40 individual LRs.

(e) Representative images and GSA distributions of Roscovitine treated Col-0 wild-type seedlings. Kolmogorov-Smirnov test P-values: *** $P < 0.001$ (compared to DMSO solvent control). Mean ± SEM, n = 5 plates (16 seedlings with 90-130 LRs per plate). Scale bars, 2 mm.

(f)-(g) Gravitropic growth rates to quantitatively asses gravitropic response of dark grown (f) *Col-0* and *CDKB1;1 DN* as well as (g) *Col-0* and *cdkb1;1 ckdb1;2*. Mean ± SD, n = 10-15 individual roots.

(a)-(g) Experiments were repeated at least three times.

(h) Schematic model depicts spatially defined gravitropic and anti-gravitropic hormonal cues at opposing organ flanks. Cytokinin signalling functions as an anti-gravitropic growth regulator at the upper side and thereby counterbalances auxin-dependent gravitropic growth of lateral roots.

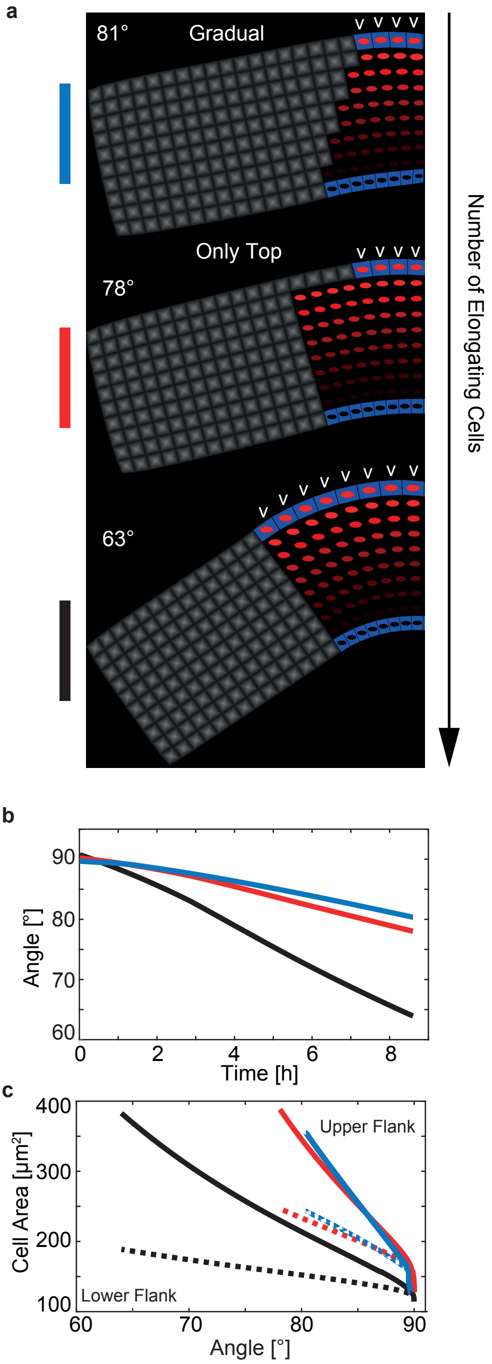

Supplementary Figure 9. Simulations of dynamic computer model addressing the gradual decrease of cell numbers along a lateral root organ.

(a) Simulated reduction of elongating cells, which are either restricted to the upper part (red) or gradually extent from the bottom towards the top (blue), induce reduced curvature compared to the control scenario (black).

(b) Time evolution of set-point angle corresponding to respective scenarios.

(c) Predicted GSA angle plotted against the predicted cell size. Dashed and filled lines depict lower and upper root flank, respectively. Colours in (b) and (c) correspond to (a). Simulations represent LR status after 8h of dynamic elongation.
