# Supplementary material for: Cytokinin functions as an asymmetric and anti-gravitropic signal in lateral roots
Source: Nat Commun. 2019 Aug 6;10:3540. doi: 10.1038/s41467-019-11483-4 (PMC6684572; doi:10.1038/s41467-019-11483-4)
Supplement: Supplementary file 3 — Description of Additional Supplementary Files [file 41467_2019_11483_MOESM3_ESM.pdf]

Description of Additional Supplementary Files

File Name: Supplementary Data 1
Description: Accessions with their mean GSA distribution (compared to Col-0) used in this study.

File Name: Supplementary Data 2
Description: All GSA distributions presented in this work.

File Name: Supplementary Data 3
Description: Oligonucleotides used in this study.
