# Supplementary material for: Cytokinin functions as an asymmetric and anti-gravitropic signal in lateral roots
Source: Nat Commun. 2019 Aug 6;10:3540. doi: 10.1038/s41467-019-11483-4 (PMC6684572; doi:10.1038/s41467-019-11483-4)
Supplement: Supplementary file 7 — Reporting Summary [file 41467_2019_11483_MOESM7_ESM.pdf]

nature research

Corresponding author(s): Jürgen Kleine-Vehn

Reporting Summary

Nature Research wishes to improve the reproducibility of the work that we publish. This form provides structure for consistency and transparency in reporting. For further information on Nature Research policies, see Authors & Referees and the Editorial Policy Checklist.

Statistical parameters

When statistical analyses are reported, confirm that the following items are present in the relevant location (e.g. figure legend, table legend, main text, or Methods section).

n/a	Confirmed	
☐	☒	The exact sample size (*n*) for each experimental group/condition, given as a discrete number and unit of measurement
☐	☒	An indication of whether measurements were taken from distinct samples or whether the same sample was measured repeatedly
☐	☒	The statistical test(s) used AND whether they are one- or two-sided<br>*Only common tests should be described solely by name; describe more complex techniques in the Methods section.*
☐	☒	A description of all covariates tested
☐	☒	A description of any assumptions or corrections, such as tests of normality and adjustment for multiple comparisons
☐	☒	A full description of the statistics including central tendency (e.g. means) or other basic estimates (e.g. regression coefficient) AND variation (e.g. standard deviation) or associated estimates of uncertainty (e.g. confidence intervals)
☐	☒	For null hypothesis testing, the test statistic (e.g. *F*, *t*, *r*) with confidence intervals, effect sizes, degrees of freedom and *P* value noted<br>*Give P values as exact values whenever suitable.*
☒	☐	For Bayesian analysis, information on the choice of priors and Markov chain Monte Carlo settings
☒	☐	For hierarchical and complex designs, identification of the appropriate level for tests and full reporting of outcomes
☒	☐	Estimates of effect sizes (e.g. Cohen's *d*, Pearson's *r*), indicating how they were calculated
☐	☒	Clearly defined error bars<br>*State explicitly what error bars represent (e.g. SD, SE, CI)*

Our web collection on statistics for biologists may be useful.

Software and code

Policy information about availability of computer code

Data collection	*Provide a description of all commercial, open source and custom code used to collect the data in this study, specifying the version used OR state that no software was used.*
Data analysis	GWAS analysis was performed online: gwas.gmi.oeaw.ac.at<br>Western Blots were quantified using the Odyssey Imagine System (LI-COR).<br>Statistical analysis were performed using Graphpad Prism Version 7 or Kolmogorov-Smirnov test online (http://www.physics.csbsju.edu/stats/KS-test.n.plot_form.html)<br>The root model was created using a version of the VV simulator embedded in the modeling software L-studio (http://algorithmicbotany.org/lstudio).

For manuscripts utilizing custom algorithms or software that are central to the research but not yet described in published literature, software must be made available to editors/reviewers upon request. We strongly encourage code deposition in a community repository (e.g. GitHub). See the Nature Research guidelines for submitting code & software for further information.

Data

Policy information about availability of data

All manuscripts must include a data availability statement. This statement should provide the following information, where applicable:

- Accession codes, unique identifiers, or web links for publicly available datasets
- A list of figures that have associated raw data
- A description of any restrictions on data availability

The GWAS results can be viewed interactively online: https://gwas.gmi.oeaw.ac.at/#/analysis/12722/overview

Field-specific reporting

Please select the best fit for your research. If you are not sure, read the appropriate sections before making your selection.

[x] Life sciences [ ] Behavioural & social sciences [ ] Ecological, evolutionary & environmental sciences

For a reference copy of the document with all sections, see nature.com/authors/policies/ReportingSummary-flat.pdf

Life sciences study design

All studies must disclose on these points even when the disclosure is negative.

Sample size	No statistical methods were used to predetermine sample size, as sample size was based on availability of resources and on the number of sample repeats required to obtain definitive results.
Data exclusions	No data were excluded from the paper.
Replication	Experiments were replicated two to eight times, and the results were very similar and reproducible. Simulation results were also reliably reproducible.
Randomization	Experimental plant materials were collected randomly without any bias.
Blinding	The experimental materials are plants (seedlings or adult plants), thus the blinding design is not applicable to this system.

Reporting for specific materials, systems and methods

Materials & experimental systems

n/a	Involved in the study	
[ ]	[x]	Unique biological materials
[ ]	[x]	Antibodies
[x]	[ ]	Eukaryotic cell lines
[x]	[ ]	Palaeontology
[x]	[ ]	Animals and other organisms
[x]	[ ]	Human research participants

Methods

n/a	Involved in the study	
[x]	[ ]	ChIP-seq
[x]	[ ]	Flow cytometry
[x]	[ ]	MRI-based neuroimaging

Unique biological materials

Policy information about availability of materials

Obtaining unique materials	Plasmid constructs and plant lines used in the study are available from the authors upon request.

Antibodies

Antibodies used	α-GFP, abcam, ab290, Lot #GR76757-1; α-Tubulin, Sigma, T6074, Lot #037M4804V
Validation	All antibodies used were validated by western blot with purified protein from Arabidopsis materials,
